# Molecular Binding Interactions: Their Estimation and Rationalization in QSARs in Terms of Theoretically Derived Parameters

**DOI:** 10.1100/tsw.2002.343

**Published:** 2002-06-27

**Authors:** David F.V. Lewis, Howard B. Broughton

**Affiliations:** Molecular Toxicology Group, School of Biomedical and Life Sciences, University of Surrey, Guildford, Surrey, GU2 7XH, UK; Lilly S.A., Avda de la Industria 30, 28108 Alcobendas, Madrid, Spain

**Keywords:** drug-receptor binding interactions, quantitative structure-activity relationships (QSARs)

## Abstract

An extensive survey of molecular binding interactions and parameters used in QSARs is reported, which includes consideration of lipophilicity and the derivation of Linear Free Energy Relationships associated with drug-receptor binding, together with an overview of the various contributions to binding energy. The lipophilic parameter, log P, and its relevance to desolvation energy is outlined and explanation of the parameters derived from electronic structure calculation is provided, leading into a summary of molecular dynamics simulations.

## INTRODUCTION

In the 1890s, Meyer[1] and Overton[2] noted that narcosis of anaesthetics could be related to oil-water partition coefficients. This finding was extended by Ferguson[3] and Collander[4], who showed that such relationships could be expressed quantitatively; but by far the most important and significant advances in the QSAR field were made by Hansch and coworkers[5,6,7,8] when it was demonstrated that the logarithm of the octan-1-ol water partition coefficient could be linearly related to biological activity in many series of compounds using equations of the type:

$$\log A = a \log P + b \quad (1)$$

where A and P are the biological activity and partition coefficient, respectively, and a and b are constants for a given series of compounds.

Based on the logarithm of experimentally determined values of partition coefficients, Hansch, Leo, and coworkers[9,10] formulated the hydrophobic substituent parameter, π, where:

$$\pi_{\text{substituent}} = \log P_{\substack{\text{substituted}\\ \text{compound}}} - \log P_{\substack{\text{parent}\\ \text{compound}}} \tag{2}$$

One of the important outcomes of this work was that the hydrophobic effect of molecular fragments could be quantified in such a way that contributions to ligand-receptor or substrate-enzyme binding energies made by hydrophobic interactions could be estimated from experimentally measurable partition coefficients. An alternative approach has been to measure the thermodynamics of phase transfer processes for various compounds from aqueous to nonaqueous environments[11]. More recently, Andrews and coworkers[12] have extended this to study protein interactions in more detail from an investigation of drug-receptor binding energies.

## DERIVATION OF THE p$^2$-p-s EQUATION

The Hansch group recognized the utility of the Hammett substituent parameter, σ, in estimating the contribution from electronic factors in the formulation of biological QSARs. Moreover, it was also found that, in certain instances, a quadratic equation in the hydrophobic parameter, π, gave rise to an improved correlation with some forms of biological activity. These two inclusions, which produce a more widely applicable linear free-energy relationship (LFER) methodology to QSAR analysis, may be derived from a consideration of structural contributions to biological activity according to the following procedure.

One form of mathematical expression which has been used to relate biological activity with concentration of the effector chemical is based on the assumption that the rate of biological response (dR/dt) can be equated with the product of three factors: the effector concentration ($C_x$), the probability of the effector reaching the site of action ($P_x$), and its reactivity ($K_x$), as follows:

$$\frac{dR}{dt} = P_x \cdot C_x \cdot K_x \tag{3}$$

Hansch and coworkers have demonstrated that the probability, $P_x$, can be expressed as an exponential Gaussian function in terms of π, the Hansch hydrophobic substituent parameter; that is:

$$P_x = ae^{-(\pi-\pi_o)^2/b} \tag{4}$$

where a and b are constants, e is the exponential, π is the Hansch hydrophobic substituent parameter (previously defined in Eq. 2), and $\pi_o$ is the optimal π value for transport to the active site or biological receptor.

Combining Eqs. 3 and 4 gives the following expression:

$$\frac{dR}{dt} = ae^{-(\pi-\pi_o)^2/b} C_x \cdot K_x \tag{5}$$

Now, if $C_x$ is defined as the effector dose concentration required to elicit a unit response in a given time, t, then:

$$\frac{dR}{dt} = 1 \tag{6}$$

It follows that, if we use this condition to substitute for dR/dt in Eq. 5 and then take the logarithm of both sides of the expression, we get:

$$0 = \log a - \frac{(\pi - \pi_o)^2}{b} + \log C_x + \log K_x \tag{7}$$

Rearrangement of this equation gives:

$$-\log C_x = \log a - \frac{(\pi - \pi_o)^2}{b} + \log K_x \tag{8}$$

Expansion of the term in brackets produces a quadratic equation in p, as follows:

$$-\log C_x = -k_2\pi^2 + k_1\pi + \log K_x + k_3 \tag{9}$$

where $k_1$, $k_2$, and $k_3$ are constants because $\pi_o$ is also a constant.

From the Hammett equation, chemical reactivity, $K_x$, is a linear function of the substituent parameter $\sigma$ for a given series and reaction; that is:

$$\log K_x = \rho\sigma \tag{10}$$

where $\rho$ is a constant for a given reaction.

Substitution of $\log K_x$ in Eq. 9 produces the familiar $\pi^2$-$\pi$-$\sigma$ equation, as follows:

$$-\log C_x = k_1\pi - k_2\pi^2 + \rho\sigma + k_3 \tag{11}$$

In QSAR analysis, the constant terms $k_1$, $k_2$, $k_3$, and $\rho$ are determined by multivariate methods — such as multiple stepwise regression analysis, principal components analysis, and the technique of partial least squares, for example — to produce a linear equation relating various substituent parameters with biological activity.

## THEORETICAL DESCRIPTORS OF BINDING INTERACTIONS

It is generally regarded that the rate-determining step in the production of a biological response is the formation of a substrate-receptor complex, which then elicits a response at a significantly higher velocity. Symbolically, this situation can be expressed as follows:

$$S + R \xrightarrow{k_1} SR \xrightarrow{k_2} \text{biological response}$$

where S and R refer to substrate and receptor, respectively, and SR represents the substrate-receptor complex. The rate constants for the formation of the substrate-bound complex and for the production of a response are shown as $k_1$ and $k_2$, respectively, where $k_2 >> k_1$. However, $k_2$ may in fact represent a series of processes leading to the development of a biological effect and can, for example, involve a number of stages of signal transduction that are associated with macromolecular interactions which often include charge transfer.

As the binding of a substrate, or ligand, to a receptor is a reversible process, it is possible to determine the equilibrium constant for the binding interaction. It is therefore reasonable to relate binding affinity (and, sometimes, biological activity) with the free energy of formation of the substrate-receptor complex, $\Delta G_{RS}$. Assuming that electronic, solvent, and steric interactions between substrate and receptor, and also any conformational changes in the receptor, are first order and separable, it is possible to formulate an expression for the activity, $A_n$, of a compound n in a series of N compounds tested against a common receptor, as follows:

$$A_n = \Delta G_{RS} = \Delta G_n^e + \Delta G_n^d + \Delta G_n^s + \Delta G_n^p + k \tag{12}$$

where k is a constant and the $\Delta G_n$ terms refer to electronic, desolvation, steric, and conformational contributions, respectively, to the overall free energy change.

## QSAR Rationale

If such a series of compounds differs only by substitution at various positions in a parent species, then it can be shown[13] that the change in $A_n$ through the series is paralleled by changes in substituent properties, as follows:

$$\delta A_n = \delta\left(\Delta G^e\right)_n + \delta\left(\Delta G^d\right)_n + \delta\left(\Delta G^s\right)_n + \delta\left(\Delta G^p\right)_n \tag{13}$$

Assuming that conformational changes in the receptor are approximately constant within the series, it is possible to equate Eq. 13 with the following linear free energy (LFER) equation:

$$\delta A_n = a\sigma + b\pi + cE_s + k \tag{14}$$

where a, b, c, and k are constants, $\sigma$ is the Hammett (electronic) substituent parameter, $\pi$ is the Hansch (lipophilic/hydrophobic) substituent parameter relating to desolvation events occurring during the formation of the substrate-receptor complex, and $E_s$ is the Taft (steric) substituent parameter. Eq. 14 enables one to account for biological activity changes within a series of structurally related compounds in terms of the linear free energy parameters derived by Hansch analysis (see Eq. 11).

For the purpose of a theoretical rationalization for the use of quantum-chemical parameters in QSAR analysis, Cammarata[14] found it convenient to express Eq. 12 in terms of contributions from electronic, desolvation, and steric effects between interacting atoms on the substrate, s, and receptor, r, as follows:

$$A_n = \sum_r \sum_s \left(E_{rs} + L_{rs} + V_{rs}\right)_n + k \tag{15}$$

where $E_{rs}$ is the electronic component, $L_{rs}$, is the lipophilic (or desolvation) component, $V_{rs}$ is the volume (or steric) component, and k is a constant. The summations (n) are over all atoms comprising the receptor molecule (r) and ligand or substrate (s).

For a series of structurally related molecules, $E_{rs}$, $L_{rs}$, and $V_{rs}$ can be assumed to be constant for atoms that are common to each compound, leading to the following reduction:

$$A_n = \sum_g a_{ng} + c \tag{16}$$

where c is a constant and $a_{ng} = \sum_r \sum_s (E_{r's'} + L_{r's'} + V_{r's'})$. The primes indicate that specific interactions are identified with a given atom or group, g. Eq. 16 forms the basis of Free and Wilson's approach to structure-activity correlations[15].

## Theoretical Rationale

The $E_{rs}$ term in Eq. 15 can be expressed in terms of theoretically derived parameters obtained from electronic structure calculations using the expression derived by Klopman and Hudson[16,17,18] based on the assumption that interactions between substrate and receptor are associated with electron transfer. The quantum-chemical formulation of the electronic contribution then becomes:

$$E_{rs} = \frac{-q_r q_s e^2}{\varepsilon_{rs} D_{rs}} + \sum_m^{occ} \sum_n^{unocc} \frac{2C_{nr}^2 C_{ns}^2 \beta^2}{E_m - E_n} \tag{17}$$

where $q_r$ and $q_s$ are net electronic charges on the receptor and substrate atoms, respectively, separated by a distance, $D_{rs}$, in a medium of dielectric constant, $\varepsilon_{rs}$; e is the charge on the electron; and $C_{mr}$ and $C_{ns}$ are the molecular orbital (MO) coefficients for receptor and substrate molecules, respectively, with orbital energies $E_m$ and $E_n$. These energies may be approximately equated with the LUMO and HOMO energies, as the $E_m$-$E_n$ value will be small, and the summations are over occupied and unoccupied MOs, whereas $\beta$ is the resonance integral obtained from the MO formalism.

When $E_m - E_n >> 0$, the second term in Eq. 17 becomes negligible compared with the first (coulombic) term, and the overall interaction is largely dependent on the charges $q_r$ and $q_s$. This gives rise to what is known as a *charge-controlled interaction,* where Eq. 17 becomes:

$$E_{rs} = \frac{-q_r q_s e^2}{e_{rs} D_{rs}} + 2\left(\sum_m C_{mr}^2\right)\left(\sum_n C_{ns}^2\right)\gamma \tag{18}$$

where $\gamma = \beta^2/(E_m - E_n)$. In this case, the main contribution to $E_{rs}$ will be the coulombic term because the second term is small in comparison.

However, when $E_m \cong E_n$* the second term in Eq. 17 becomes predominant, giving rise to a so-called *frontier-controlled interaction.* This is due to the fact that it is the frontier orbital electrons (i.e., those associated with the frontier orbitals, HOMO and LUMO) that are involved in the interaction. In this case, the expression for $E_{rs}$ becomes:

$$E_{rs} = 2C_{mr}C_{ns}\beta \tag{19}$$

* The derivation of Eq. 17 assumes that $E_m$ and $E_n$ will not be exactly equal, however, as this would make the second term infinite.

such that the electronic reactivity is determined by the frontier orbital electron density, $f_{ns}$, which is proportional to the corresponding orbital coefficients. Cammarata has reported examples where either net atomic charges or frontier electron densities correlate with biological activity, thus demonstrating that electronic effects can make major contributions to substrate-receptor binding[19]. Furthermore, Cammarata has shown that an orbital-weighted electron density parameter, known as *superdelocalizability* ($S_E$), can be incorporated into the frontier orbital term of Eq. 17, which may then be rewritten as:

$$E_{rs} = \frac{-q_r q_s e^2}{e_{rs} D_{rs}} + 1/2 q_r S_E \beta \qquad (20)$$

The electrophilic superdelocalizability, $S_E$, is a term previously used by Fukui and coworkers[20] as a reactivity index, where:

$$S_E = 2\sum_m^{occ} \frac{C_{ms}^2}{E_m} \qquad (21)$$

This parameter, which can be calculated by MO procedures, was originally derived by Fukui et al.[20]. If electronic charges associated with the receptor are negligible, but overall electronic charges within a series of structurally related substrates relate to activity differences, it is reasonable to simplify Eq. 20 to the following:

$$A_n = a_s \sum \left( a q_s + b S_E \right) + c \qquad (22)$$

where a, b, and c are constants obtained from correlation analysis. Cammarata has demonstrated that the use of such parameters, derived from MO calculations, can produce statistically significant QSARs in certain series of compounds. Moreover, the same author advanced a theoretical expression for lipophilicity by equating the Hansch hydrophobic substituent parameter, $\pi$, with electric polarizability, $\alpha$, a parameter which can be calculated by MO procedures or obtained from experimental measurements[14,19]. The hydrophobic parameter, log P, correlates with polarizability in series of nonpolar compounds, but additional terms governing electric dipole moment and electron-donor ability are required in order to describe fully the variations in log P for structurally diverse chemicals[21]. Furthermore, Cammarata has also shown previously that both $\pi$ and log P values relate well with a linear combination of net atomic charge, $Q_T$, and superdelocalizability, $S_E$, summed over all atoms in the molecule (or group), using equations of the type:

$$\log P \left( \text{or } \pi \right) = a_s \sum Q_T + b_s \sum S_E \qquad (23)$$

where, in some cases, either or both of these electronic components correlate(s) with activity data in a variety of biological systems[22].

The hydrophobic parameter, log P, and its associated substituent parameter, $\pi$, have been utilized in the generation of a large number of QSARs for many types of biological activity. However, the component nature of log P has prompted some workers to investigate the possibility of expressing this physiochemical parameter in terms of either experimentally derived[23,24] or theoretically derived[21] parameters. QSARs also provide relative estimates of important quantities such as binding energies, but it may sometimes be preferable to derive absolute values for the energies involved in binding interactions. This aspect is discussed in the following section.

## INTERACTION ENERGIES IN BIOLOGICAL SYSTEMS

One goal of QSAR analysis is to obtain a description of activity changes towards a given biological endpoint, for a series of structurally related chemicals, in terms of a number of experimental or theoretical parameters, which relate to known properties, such that some degree of rationalization is possible with respect to molecular interactions between the compounds and a biological receptor. It is possible to make estimations of the binding energies due to biomolecular interactions in one of three ways: by approximating the contributions to the overall thermodynamic changes which occur during such processes; by calculating values of each component of the binding energies; or by considering the results of QSAR analysis using physico-chemical and/or quantum-chemical measurements. There is some degree of equivalence between the classical QSAR parameters, such as log P, π, σ, and others, and quantum mechanical parameters calculated via MO procedures. However, this comparability is rarely sufficiently precise to merit the replacement of one type by another; consequently, both are extensively used by many workers in the field.

In the previous section, it has been shown how it is possible to express overall biological activity in terms of a number of components pertaining to specific interaction energies. We have also seen that a linear combination of substituent parameters can relate to bioactivity via the free energy changes ($\Delta G_n$) in certain relevant processes: this is the origin of the term *linear free energy relationship* (LFER). LFERs have also been utilized to describe chemical reactivities and properties, as well as in the description of biological activities and related processes. LFERs can, therefore, be regarded as a subset of QSARs and the associated quantitative structure-property relationships (QSPRs), which are concerned with quantitative descriptions of physico-chemical properties in terms of various structural parameters.

Biological activity can be the result of covalent bond formation or fission, both homolytic and heterolytic, which give rise to either free radicals or ions, respectively; or it can involve one or more type(s) of intermolecular interactions, such as electrostatic (ionic), dipolar, charge transfer, hydrogen bonding, hydrophobic, and van der Waals interactions; the last is composed of a combination of permanent, induced, and instantaneous dipole-dipole interactions. Each of these forms of intermolecular interactions will now be discussed in some detail.

### Electrostatic Interactions

The energy of electrostatic attraction, ΔE, between two ionic charges, $q_1$ and $q_2$, is governed by Coulomb's Law, which can be expressed as:

$$\Delta E = \frac{q_1 q_2 e^2}{\varepsilon d} \qquad (24)$$

where e is the electronic charge ($1.6 \times 10^{-19}$ coulomb) and ε is the dielectric constant of the medium separating the charges at a distance, d. For oppositely charged species, the electrostatic interaction energy expressed in $kcal.mol^{-1}$ is given by the relationship:

$$\Delta E = \frac{-332 q_1 q_2}{\varepsilon d} \qquad (25)$$

where the ionic separation distance, d, is given in Ångstrom units ($1\ Å = 10^{-10}$ m). Thus, for two unit ionic charges at a distance of 4Å in an aqueous medium (ε = 79), the interaction energy is about

1 kcal.mol$^{-1}$, which will give rise to a difference in the equilibrium constant by a factor of five at 37°C. However, when considering biomolecular interactions, it is not always valid to assume that the dielectric constant will be the same as that of pure water. This is because an essentially hydrophobic environment can exist between interacting charged species once desolvation has occurred, and this is a common situation in biophase reactions. Such an occurrence will considerably reduce the local dielectric constant by a factor of at least twentyfold in some circumstances. The precise value of the local dielectric constant is usually difficult to calculate, but it can often be expressed as a distance-dependent function.

Internal ion pairs in protein structures, where there is little or no intervening solvent, are quite strong (–4 to –8 kcal.mol$^{-1}$) and assist in the folding of the peptide backbone into a tertiary structure, whereas external ionic residues on the surface of proteins are responsible for protein aggregation, aqueous solubility, and protein recognition interactions, with some of these possibly leading to electron transfer processes.

## Dipolar Interactions

There are four forms of dipolar interactions of relevance to substrate-receptor associations. These are: dipole-dipole, ion-dipole, ion–induced dipole and dipole–induced dipole interactions. As the energy terms and ranges are slightly different in each case, these will be discussed under the following subheadings.

### *Dipole-Dipole*

The interaction energy, $\Delta E$, between two dipoles, $\mu$ and $\mu_2$, orientated at an angle $\theta$ with an interdipolar distance, d, in a medium of dielectric constant, $\varepsilon$, is given by the expression:

$$\Delta E = -\frac{\mu_1\mu_2 \cos\theta}{\varepsilon d^3} \qquad (26)$$

For dipole moments expressed in debyes (where 1 debye = 3.336 × 10$^{-30}$ C.m.) and inter-dipole distances in Ångstrom units (1 Ångstrom = 10$^{-10}$ m.), Eq. 26 can be rewritten as:

$$\Delta E = \frac{14.4\mu_1\mu_2 \cos\theta}{\varepsilon d^3} \qquad (27)$$

where the energy is given in kcal.mol$^{-1}$. Eq. 27 facilitates the calculation of dipolar interactions between permanent dipoles. The inverse cubic relationship between distance and dipolar interaction energy indicates that a closer approach than that needed for ionic attraction is necessary for a strong interaction to occur, and that dipole-dipole forces are generally weaker than electrostatic forces.

Hydrogen bonding can be considered as a special case of dipole-dipole interaction that is usually stronger due to the relatively short distance ranges (2.5 to 3.5 Å) and angle constraints brought about as a result of the directional nature of nonbonding (lone pair) electron orbitals. The energy range for dipole-dipole interactions is about –3 to –5 kJ.mol$^{-1}$ or between –0.7 and –1.2 kcal.mol$^{-1}$, whereas hydrogen bonding energies lie between –8 and –32 kJ.mol$^{-1}$, corresponding to between –2 and –8 kcal.mol$^{-1}$, although lower values have been reported in some cases[25]. As a particular example, the dipolar energy between tyrosine and camphor at an orientation found in the active site of cytochrome $P450_{cam}$ (the enzyme which specifically mediates camphor 5-hydroxylation) can be calculated to be between –3.5 and –4.6 $kcal.mol^{-1}$ depending on whether the experimental or calculated dipole moments are employed. The formation of this hydrogen bond is thought to make a significant contribution to the substrate binding energy of about –7 $kcal.mol^{-1}$. Dipole-dipole interactions, being highly directional, are partially responsible for the base-paired stacking energies in nucleic acids, for example[26].

## Ion-Dipole

For an ion of charge, q, interacting with a dipole, μ, at an angle, θ, and distance, d, in a medium of effective dielectric constant, e, the interaction energy is given by:

$$\Delta E = -\frac{q\mu \cos\theta}{\varepsilon\left(d^2 - r^2\right)} \tag{28}$$

where r is the length of the dipolar bond. The force of this attraction (or repulsion, depending on orientation) is intermediate between electrostatic and dipole-dipole interactions. For interactions between ions and dipoles (where dipole moments are given in debyes) at distances measured in Ångstrom units, the energy expressed in $kcal.mol^{-1}$ becomes:

$$\Delta E = \frac{69.1\,\mu \cos\theta}{\varepsilon\left(d^2 - r^2\right)} \tag{29}$$

One common example of ion-dipole interaction is the hydration of ions in aqueous solution. Such interactions also occur between the surface charges of proteins and bulk water. However, both ion-dipole and dipole-dipole associations are found extensively in biological systems, particularly in enzyme-substrate and drug-receptor interactions.

## Ion–Induced Dipole

The electric field of an ion can induce a dipole in a nonpolar molecule and thus give rise to an interaction similar to that discussed previously. In this case, the energy expression involves an inverse function with respect to the ion-molecular distance, which is of the type:

$$\Delta E = -\frac{q^2\alpha}{\varepsilon^2 d^4} \tag{30}$$

where α is the polarizability of the molecule wherein a dipole charge separation is ion induced. The ionic charge, q, is at a distance, d, from the molecule in a medium of dielectric constant, ε. Conversion of this expression, shown in Eq. 30, to give the interaction energy in $kcal.mol^{-1}$ for polarizabilities measured in cubic Ångstroms, gives:

$$\Delta E = -\frac{166q^2\alpha}{\varepsilon^2 d^4} \tag{31}$$

for distances in Ångstrom units between the ion and polarized molecule. Ion-induced dipole interactions are associated with energies that are relatively small compared with those discussed previously, being of the order of about –0.5 kcal.mol$^{-1}$, and require a closer approach of interacting species than is necessary for electrostatic and dipolar forces.

## *Dipole–Induced Dipole*

This is another form of weak, short-range intermolecular force, and its energy expression is related to that described in the previous example. The force arises from the induction of a dipole in a nonpolar, but polarizable, molecule due to the close approach of another molecule with a permanent dipole moment. The energy for such an interaction is given by the expression:

$$\Delta E = \frac{-\mu^2 \alpha \left(1 + 3\cos^2\theta\right)}{\varepsilon^2 d^6} \tag{32}$$

where $\mu$ is the permanent dipole moment, $\alpha$ is the polarizability of the dipole-induced molecule, d is the distance between the two molecules, and $\varepsilon$ is the dielectric constant of the intervening medium. Induction of a dipole in the polarized molecule produces a dipole-dipole interaction between the two molecules, where $\theta$ is the orientation angle between their respective dipoles. The inverse relationship between the energy and the sixth power of the distance shows that the forces will be weak and highly distance dependent. For mutually aligned molecules, which ensure a favourable interaction, the energy expression that provides dipole–induced dipole interaction energies in kcal.mol$^{-1}$ is as follows:

$$\Delta E = \frac{-14.4\mu^2 \alpha}{\varepsilon^2 d^6} \tag{33}$$

where the dipole moment is measured in debyes, polarizability is in cubic Ångstroms, and distance is in Ångstrom units. Dipole-induced dipole interactions are common in substrate-receptor interactions but are usually small enough to be negligible for most applications. The usual energies are normally about –0.5 kcal.mol$^{-1}$, which is roughly the same as for ion-induced dipolar interactions.

## Charge-Transfer Interactions

Charge-transfer (CT) or electron-transfer complexes are formed by the transfer of charge from one molecule (the electron donor, D) to another (the electron acceptor, A). The overall energy, $\Delta E$, for this type of interaction is given by the following expression:

$$\Delta E = \frac{E^2}{I_D - E_A - q^2/d} \tag{34}$$

where E is the actual donor-acceptor interaction energy between the two species, $I_D$ is the ionization energy of the donor species, $E_A$ is the electron affinity of the acceptor molecules, q is the charge transferred, and d is the distance between the two species which constitute the donor-acceptor charge-transfer complex. The donor-acceptor interaction energy E, which is associated with the interaction process between the two molecules that precedes the transfer of charge, can be calculated using a quantum-mechanism expression involving the overlap between HOMO and LUMO orbitals on donor and acceptor molecules, respectively. Charge-transfer energies are roughly of the same energy and distance ranges as those for hydrogen bonded interactions: between –2 and –9 kcal.mol$^{-1}$ and, on average, around 3Å apart.

## Van der Waals Interactions

These weak and short-range interactions are also sometimes described as *London* (or *dispersion*) forces and arise from a summation of the instantaneous dipole fluctuations brought about by the motion of electrons relative to the positive nuclei of atoms in molecules, which induces dipoles in proximate species. In his original description, van der Waals included other forms of dipolar attraction as described previously. However, it appears that an appropriate expression for the attraction energy, ΔE, between two species is given by:

$$\Delta E = \frac{-1.5\alpha_1\alpha_2}{d^6}\left(\frac{I_1 I_2}{I_1 + I_2}\right) \tag{35}$$

where $\alpha_1$ and $\alpha_2$ are the polarizabilities of groups 1 and 2 with ionization energies $I_1$ and $I_2$, respectively, separated by a distance d. For polarizabilities in Å$^3$, distances in Å, and ionization energies in kcal.mol$^{-1}$, the expression becomes:

$$\Delta E = \frac{-17.3\alpha_1\alpha_2}{d^6}\left(\frac{I_1 I_2}{I_1 + I_2}\right) \tag{36}$$

Although individually weak in comparison with other intermolecular interactions, van de Waals forces become significant at small distances, and in situations where there is a relatively large number of similar groupings in close proximity. For example, in the case of methylene groups in close contact, the van der Waals attraction energies increase approximately with the square of the number of groups in a hydrocarbon chain, i.e., –0.095, –0.38, and –0.86 kcal.mol$^{-1}$, respectively, for 1, 2, and 3 bonded methylene groups in parallel chains. It is probable, therefore, that van der Waals forces make an important contribution to the attractions between phospholipid molecules in a cell membrane, and also in drug-receptor binding interactions where there is a close fit between interacting species. The usual range of these energies is between –0.1 and –1 kcal.mol$^{-1}$, depending on the nature of the interaction, and Table 1 summarizes the various expressions for the above types of intermolecular interaction energies, together with those relating to hydrophobicity/lipophilicity as described below.

## Hydrophobic Interactions

The positive entropy change (termed the *hydrophobic effect*) that occurs when hydrocarbons associate in aqueous solution is thought to be due to the increase in disorder of water molecules transferred from the hydrocarbon surface to the bulk solvent environment[27]. Thermodynamic aspects of the hydrophobic effect have been described by Taylor[28], and the Appendix provides one example of how a theoretical treatment can be employed to describe the hydrophobicity (or lipophilicity) of a compound in terms of the $\log P_{oct}$ value.

Various estimates have been made for the overall entropy increase associated with the hydrophobic effect (reviewed in Sharp et al.[29]), although the previously accepted value of 25 cal.mol$^{-1}$/Å$^2$ has been revised upwards to a figure of 47 cal.mol$^{-1}$/Å$^2$, based on a careful analysis of microscopic and macroscopic hydrophobic effects[29]. This indicates that there is compelling evidence for the latter value being more appropriate in systems operating at physiologically relevant temperatures (i.e., around 310 K). Consequently, an estimate of the hydrophobic contribution to the overall binding energy can be made on the basis of a calculation of the hydrocarbon

**TABLE 1**
**Expressions for Intermolecular Interactions Corrected for Short-Range Repulsions**

| Interaction | Normal Equation | Correction Factor | Corrected Equation |
|---|---|---|---|
| Ion-ion | $\frac{-331.9}{rD} q_1 q_2$ | 0.92 | $\frac{-304.2}{rD} q_1 q_2$ |
| Ion-dipole | $\frac{-69.1}{r^2 D} q\mu \cos\theta$ | 0.83 | $\frac{-57.6}{r^2 D} q\mu \cos\theta$ |
| | $\frac{-80.1 \times 10^4}{r^4 D^2 T} q^2 \mu^2$ | 0.67 | $\frac{-53.4 \times 10^4}{r^4 D^2 T} q^2 \mu^2$ |
| | $\frac{-14.3A}{r^3 D} \mu_1 \mu_2$ | 0.75 | $\frac{-10.7A}{r^3 D} \mu_1 \mu_2$ |
| Dipole-dipole | $\frac{-3.5 \times 10^4 \mu_1^2 \mu_2^2}{r^4 D^2 T} \left(1 + 3\cos^2\theta\right)^{1/2}$ | 0.5 | $\frac{-1.75 \times 10^4 \mu_1^2 \mu_2^2}{r^6 D^2 T} \left(1 + 3\cos^2\theta\right)^{1/2}$ |
| | $\frac{-7.0 \times 10^4}{r^6 D^2 T} \mu_1^2 \mu_2^2$ | 0.5 | $\frac{-3.5 \times 10^4}{r^6 D^2 T} \mu_1^2 \mu_2^2$ |
| Ion-induced dipole | $\frac{-165.2}{r^4 D^2} q^2 a$ | 0.67 | $\frac{-110.1}{r^4 D^2} q^2 a$ |
| Dipole-induced dipole | $\frac{-7.17 a\mu^2}{r^6 D^2} \left(1 + 3\cos^2\theta\right)$ | 0.5 | $\frac{-3.59 a\mu^2}{r^6 D^2} \left(1 + 3\cos^2\theta\right)$ |
| | $\frac{-14.34}{r^6 D^2} a\mu^2$ | 0.5 | $\frac{-7.17}{r^6 D^2} a\mu^2$ |

Reference:[40].

surface area of the molecule that is solvent-accessible. In this respect, the hydrophobic contribution can be referred to as a desolvation energy (or entropy), as it represents the quantity of solvent (i.e., water) molecules displaced upon ligand binding to a protein, for example. In terms of methylene groups, it has been estimated that the hydrophobic effect is about –0.7 kcal.mol$^{-1}$ for every pair of –$CH_2$-groups combining to the exclusion of aqueous solvent[30]. In this context, there is a clear relation between the hydrophobic interaction and van der Waals association, as described in the previous section.

## QSAR PARAMETERS: A QUANTUM-MECHANICAL DESCRIPTION

### The Component Nature of log P

Although highly successful in describing many areas of structure-activity analysis, log P does not always precisely describe the link between chemical structure and biological activity. This is due to the fact that log P comprises a number of components that relate to various aspects of molecular structure.

For example, it can be shown[22] that log P is linearly related to the energy of partitioning by the equation:

$$\log P = \frac{\Delta E_{part}}{2.3RT} + \text{constant} \quad (37)$$

where R = gas constant and T = absolute temperature.

The partitioning energy, $\Delta E_{part}$, is the difference between the solvation energy, $\Delta E_{solv}$, of a compound in aqueous and organic phases, i.e.:

$$\Delta E_{part} = E_{solv(aq)} - E_{solv(org)} \quad (38)$$

The solvation energy is, in turn, made up of a number of components (see Appendix) of which the most important are the electrostatic and polarization energy changes, as follows:

$$E_{solv} = \Delta E_{el} + \Delta E_{pol} \quad (39)$$

where the polarization energy is a linear function of polarizability, $\alpha$. Cammarata and Rogers[22] have shown that the polarization energy term is related to Fukui's superdelocalizability indices. Electrophilic superdelocalizability, $S_E$, for example, is expressed as follows:

$$S_E = 2\sum_i \frac{c_i^2}{E_i} \quad (40)$$

where i denotes occupied orbitals with coefficients $c_i$ and energies $E_i$, which may be compared with the quantum-mechanical expression for polarizability as shown below:

$$\alpha = -4\sum_i \sum_j \frac{c_i^2 c_j^2}{\left(E_i E_j\right)} \quad (41)$$

where i and j denote occupied and unoccupied orbitals, respectively, with coefficients c and energies E.

Therefore, when we find correlations with $\alpha$ (or with superdelocalizability) and biological activity, we are highlighting the important component of log P (i.e., the polarization term) which adequately explains the biological data. In addition, the solvation energy model has been employed for calculating blood-brain partitioning of organic solutes, showing a correlation of 82% for 55 compounds in the training set[31]. Consequently, the application of such theoretical models to biological events can yield important results for aiding future drug screening programmes.

## Explanation of Electronic Parameters Used in QSAR Expressions

### *Dipole Moment, m*

This quantity is the resultant dipole of a molecule brought about from contributions of individual bond dipoles. As calculated from electronic structure determinations, such as those involving MO methods, the dipole moment is obtained from considerations of individual partial atomic charges brought about by electronic distributions within the molecule. The dipole moment of simple linear diatomic molecules is defined as the product of charge separation and interatomic distance:

$$\mu = q \cdot d \tag{42}$$

where q = charge and d = distance. The units of dipole moments are debyes (D.) where 1 debye = $3.33 \times 10^{-30}$ coulomb.meter.

The magnitude of the dipole moment is a description of the degree of polarity of the molecule and, as such, is an important quantity in intermolecular interactions, including those associated with hydrogen-bond formation and other related dipole-dipole and ion-dipole interactions. Being related to polarity, the dipole moment is a component of the partitioning energy, which is directly proportional to log P, where P is the partition coefficient between aqueous and nonaqueous phases.

### *Polarizability, a*

The molecular polarizability gives an indication of the ability of a molecule to form a dipole, i.e., to polarize, under the influence of an external electric field, which may occur in certain environments such as in the vicinity of dipolar and charged species. This quantity is also calculable by MO techniques, where it is obtained from a summation of contributions from individual atomic polarizabilities. As is the case with dipole moments, molar polarizabilities are experimentally measurable quantities and may also be calculated from refractive indices. The molecular polarizability calculated via MO procedures is obtained as follows:

$$\alpha = -4\sum_i \sum_j \frac{c_i^2 c_j^2}{\left(E_i - E_j\right)} \tag{43}$$

where i and j denote occupied and unoccupied orbitals, respectively, with coefficients c and energies E.

Polarizability makes an important contribution to the partitioning between two phases; therefore, this is a major component of the hydrophobic parameter, log P. Furthermore, $\alpha$ is involved in mathematical expressions for the van de Waals interaction energy between molecules, as described previously.

### *Activation Energy, DE*

This electronic parameter is obtained from the difference between the energies of the highest occupied and lowest unoccupied MOs and its units are usually given in electron volts (eV.), i.e.:

$$\Delta E = E(\text{LUMO}) - E(\text{HOMO}) \tag{44}$$

where E(LUMO) and E(HOMO) are the energies of the highest occupied and lowest unoccupied MOs, respectively[32].

$\Delta E$ could be regarded as being comparable with the difference between electron affinity and ionization potential and, as such, represents the energy required to promote an electron from its highest occupied energy level to the lowest empty level. The $\Delta E$ parameter, as calculated from the MO description of electronic structure, is often equated with activation energy, i.e., the energy required to activate a molecule from its ground state to the first excited state, although this is an approximation. In intermolecular interactions, $\Delta E$ makes an important contribution to the energy for charge transfer processes to occur and, consequently, is of relevance to many areas involving drug-receptor interactions[32,33].

## The Collision Diameter, cd

This is roughly equivalent to the diameter of sphere traced out by the free rotation of the molecule in question about its geometric centre along three mutually orthogonal (x,y,z) axes. The collision diameter can be obtained from the van der Waals volume and is proportional to the cross-sectional area perpendicular to the main molecular plane. In order to derive collision diameter from volume of the solvent-accessible surface, it is possible to utilize the following expression, which assumes that the surface approximates to a sphere:

$$cd \cong 2\sqrt[3]{\frac{3V}{4\pi}} \tag{45}$$

where V is the volume of the Connolly surface[34].

## Molecular Electrostatic Potential, $V_{(r)}$

The electrostatic potential, $V_{(r)}$, experienced by a unitary positive charge (i.e., a proton) at a distance, r, from a molecule will be a linear combination of attraction from the electrons, and repulsion from the nuclei, of all the atoms in the molecule. This supposition is a corollary of the Hellmann-Feynman theorem, which enables some types of quantum-chemical calculation to be performed in apparently classical terms[35,36].

From Maxwell's equations, the divergence or net outward flow per unit volume of electric field, F, into a volume element must be accounted for by the presence of free charges at a density, $\rho(r)$, in that volume. Expressed in a mathematical form, this becomes:

$$\nabla \cdot F = 4\pi\rho_{(r)} \tag{46}$$

where $\nabla$ is the del operator

$$\frac{i\delta}{\delta x} + \frac{j\delta}{\delta y} + \frac{k\delta}{\delta z}[37].$$

**TABLE 2**
**Average Binding Energy Contributions in Ligand-Protein Interactions**

| Type | Typical Value ($kcal.mol^{-1}$) |
|---|---|
| Ionic (electrostatic) energy | –4.5* |
| Hydrogen bond interaction | –2.0* |
| Aromatic ($\pi$-$\pi$ stacking interaction) | –0.9 |
| Desolvation energy | –0.025 SA |
| Bond rotors† | +0.6 per rotatable bond |
| Translational and rotational† | +log $M_r$ |

* These values are subject to local variations in dielectric constant (see Table 3).
† These quantities relate to losses in molecular degrees of freedom on binding.
SA Solvent-accessible surface area ($Å^2$) of the Connolly surface based on a 1.4 Å radius solvent sphere.
$M_r$ Relative molecular mass of the ligand.
References: [34,48].

Now, the electric field is the negative of the electric potential $V_{(r)}$, i.e.:

$$F = \nabla V_{(r)} \tag{47}$$

and, therefore, combining Eqs. 46 and 47 we get the Poisson formula:

$$\nabla^2 V = 4\pi\rho_{(r)} \tag{48}$$

where $\nabla^2$ is the Laplacian operator

$$\frac{\delta^2}{\delta x^2} + \frac{\delta^2}{\delta y^2} + \frac{\delta^2}{\delta z^2} [37].$$

The charge density $\rho_{(r)}$ can be expressed as a quantum-mechanical charge distribution function and written in terms of Gaussian-type functions, i.e.:

$$\rho_{(r)} = 2\sum_{i} \sum_{\mu\nu} c_{\mu i} c_{\nu i} A_\mu (r) e^{-a_\mu r^2} A_\nu (r) e^{-a_\nu r^2} \tag{49}$$

In the INDO procedure, Slater-type orbitals (STOs) of the form $A_\mu(r)e^{-\alpha\mu}$ make up the basis set. The STOs are expanded over a limited set of Gaussian functions and, as the product of any two

Gaussian functions can be expressed in terms of a new Gaussian function, the contribution of each pair of Gaussian functions ρ(r) can be calculated separately. For example, the contribution from an S-type Gaussian function can be calculated by the following procedure. First if

$$\rho_{(r)} = e^{-\alpha r^2} \tag{50}$$

then:

$$\nabla^2 V_{(r)} = -4\pi e^{-\alpha r^2} \tag{51}$$

For an S-type orbital there is radial symmetry, so that:

$$\nabla^2 V_{(r)} = \frac{1}{r^2}\frac{\delta}{\delta r}\left(r^2 \frac{\delta}{\delta R}\right)V_{(r)} = V''_{(r)} + \frac{2}{r}V'_{(r)} \tag{52}$$

As:

$$\frac{1}{r}\frac{\delta^2}{\delta r^2}\left(rV_{(r)}\right) = V''_{(r)} + \frac{2}{r}V'_{(r)} \tag{53}$$

Substitution gives the equality:

$$\frac{1}{r}\frac{\delta^2}{\delta r^2}\left(rV_{(r)}\right) = -4\pi e^{-\alpha r^2} \tag{54}$$

Integrating twice obtains the potential $V_{(r)}$. Therefore:

$$V_{(r)} = \frac{2\pi}{\alpha r}\int_0^r e^{-\alpha r^2}\,dr + \frac{c_1}{r} + c_2 \tag{55}$$

Now, if $r = \infty$ and $V_{(r)} = 0$, or if $r = 0$ and $V_{(r)}$ = finite, then $C_1 = C_2 = 0$, and the potential function becomes:

$$V_{(r)} = \frac{2\pi}{\alpha r}\int_0^r e^{-\alpha r^2}\,dr \tag{56}$$

For small values of r:

$$\int_0^r e^{-\alpha r^2}\,dr = \frac{1}{\alpha}\left(r - \frac{r^3}{3} + \frac{r^5}{10} + \frac{r^7}{42}\cdots\right) \tag{57}$$

Therefore:

$$V_{(r)} = \frac{2\pi}{\alpha^2}\left(1 - \frac{r^2}{3} + \frac{r^4}{10} + \frac{r^6}{42}\cdots\right) \tag{58}$$

However, if r is extended to infinity, the integral becomes:

$$\int_0^r e^{-\alpha r^2}\,dr = \sqrt{\frac{\pi}{\alpha}} \tag{59}$$

And:

$$V_{(r)} = \frac{2\pi}{\alpha r}\frac{\sqrt{\pi}}{\sqrt{\alpha}} = \frac{2}{r}\left(\frac{\pi}{\alpha}\right)^{3/2} \tag{60}$$

The electrostatic potential at a point $P_{(r)}$ is therefore given by:

$$V_{(r)} = -\int \frac{\rho(r_1)}{r - r_1} + \sum_{\alpha} \frac{Z_\alpha}{r - R_\alpha} \tag{61}$$

where the first term is the electronic term and the second is the nuclear term representing the contribution from the nuclear charge distribution in the molecule of nuclei $\alpha$ at distance $R_\alpha$ with atomic number $Z_\alpha$.

In the cartesian frame of reference, r is the distance of electrons from the origin, $R_\alpha$ is the distance of nuclei from the origin, and r is the distance of the point $P_{(r)}$ from the origin. Moreover, r-$r_1$ is the distance between the electrons at $r_1$ and the point $P_{(r)}$ at r, whereas r-$R_\alpha$ is the distance between the nuclei at $R_\alpha$ and the point $P_{(r)}$ at r.

Potential field calculations can be executed at great speed by modern computers, such that a large number of molecular electrostatic potentials (MEPs) may be processed in the form of isopotential contour maps and surfaces. This technique facilitates the identification of critical receptor binding site interactive regions within substrate molecules; consequently, it is currently being extensively used in this area.

## Molecular Dynamics of Proteins

The theoretical basis of molecular dynamics simulations lies in Newton's equation relating to the force, $F_i$, acting on a particle of mass, $m_i$, which imparts an acceleration, $a_i$. This can be expressed mathematically as follows:

$$F_i = m_i a_i \tag{62}$$

It is possible to describe interactions between atoms and molecules in terms of a potential energy function, V, which is usually composed of a number of components relating to electrostatic, bond, repulsion, and dispersion energies. In this case, the force on a given particle can be calculated from the negative gradient of the energy function, that is:

$$F_i = \frac{-dV}{dr_i} \tag{63}$$

where $r_i$ is the vector representing atomic cartesian (x,y,z) coordinates and V is the potential energy function. Combining Eqs. 62 and 63, it follows that:

$$a_i = \left( \frac{-\delta V}{\delta r_i} \right) \Big/ m_i \tag{64}$$

A molecular dynamics simulation involves evaluating the positions and velocities of every atom in a given molecule at regular discrete time steps, dt, usually of the order of femtoseconds (where 1 fs = $10^{-15}$ s). One therefore needs to calculate both the force acting on each particle and its subsequent acceleration, which is approximated using Eq. 3. Although the acceleration, $a_i$, is a vector quantity, each of the components ($ax_i$, $ay_i$, and $az_i$) can be calculated according to Eq. 1 as follows:

$$ax_i = F_x / m_i \tag{65}$$

It is possible to express each component of the atomic coordinate at time t + δt as a Taylor series in terms of the previous value. For example:

$$x_i(t + \sigma t) = x_i(t) + vx_i(t)\,dt + ax_i(t)\,d_2\,t^2/2 + \cdots \tag{66}$$

And:

$$x_i(t + \sigma t) = x_i(t) - vx_i(t)\,dt + ax_i(t)\,d_2\,t^2/2 + \cdots \tag{67}$$

where $vx_i$ (t) and $ax_i$ (t) are the velocity and acceleration components, respectively.

Equations 5 and 6 are approximated by ignoring all subsequent terms after the third; then each equation (one for each end of the three positional coordinates of each atom) is solved at each time step, dt, and repeated iteratively for all the consecutive time steps in a total time interval on the order of picoseconds (1 ps = $10^{-12}$ s). The solutions for positions and velocities, respectively can be written:

$$x_i(t + \sigma t) = 2x_i(t) - x_i(t - \delta t) + f_i(t)/m_i \tag{68}$$

And:

$$v_i(t) = \left[ \left( x_i(t + \delta t) - x_i(t - \delta t) \right) \right] / 2dt \tag{69}$$

This solution is known as the *leapfrog method* or *Verlet algorithm.* The *SHAKE algorithm* is a variant of this procedure that is particularly useful for macromolecules as a larger integration step $\delta t$ is possible, such that longer simulations can be carried out for the same number of iterations. The SHAKE algorithm constrains higher frequency motions, such as those encountered when hydrogen atoms are included in the structure, thus enabling molecular dynamics simulations to be carried out on relatively large protein structures. It should be pointed out also that all of the interaction energies that can be calculated between two molecules, and thus between a protein and a ligand in the static sense, can also in principle be calculated from multiple sample conformations taken from a molecular dynamics run[38]. Consequently, although the methods used to calculate such parameters are of necessity rather approximate, they may nevertheless improve the utility of protein-ligand models for virtual screening and database mining.

## CONCLUSIONS

Consequently, it can be appreciated that it is possible to use computational procedures to study the dynamic effects of proteins as simulations of the physiological environment. In this way, it is feasible to obtain some degree of understanding of the way in which protein conformations are affected by the binding of small molecular effectors, which is the basis of most forms of biological activity. Furthermore, the individual components of binding energy can be evaluated from first principles by deriving expressions for each form of interaction. There is also some correspondence between information provided from QSAR analyses and specific molecular/electronic parameters that form part of each contributory term to the overall binding interaction energy. Therefore, these important quantities can be obtained from calculation of the relevant compound's electronic structure; moreover, the magnitude of biological effect resulting from the molecular interaction with either an enzyme or receptor protein can thus be evaluated. However, it is beneficial to generate a model of the enzyme/receptor target such that specific interactions can be estimated directly using probe ligands or substrates.

## ACKNOWLEDGEMENTS

The financial support of GlaxoSmithKline Research and Development Ltd., Merck, Sharp and Dohme Ltd., the European Union Biomed 2 programme, and the University of Surrey Foundation Fund is gratefully acknowledged by David F.V. Lewis.

## ADDITIONAL REFERENCES

Andrews, P.R. and Tintelot, M. (1990) Intermolecular forces and molecular binding. In *Comprehensive Medicinal Chemistry. Vol 4. Quantitative Drug Design.* Hansch, C., Sammes, P.G., and Talor, J.B., Eds. Pergamon, Oxford, U.K. pp. 321–347.

Arvidsson, L.-E., Karlen, A., Nordiner, V., Kenne L., Sundell, S. and Hacksell, U. (1988) Structural factors of importance for 5-hydroxy tryptaminergic activity. Conformational preferences and electrostatic potentials of 8-hydroxy-2-(di-n-propylamino)tetralin (8-OH-DPAT) and some related agents. *J. Med. Chem*. **31**, 212–221.

Atkins, P.W. (1974) *Quanta: A Handbook of Concepts*. Clarendon Press, Oxford.

Atkins, P.W. (1987) *Physical Chemistry*. 3rd ed. Oxford University Press, Oxford.

Atkins, P.W. (1991) *Quanta: A Handbook of Concepts*. 2nd ed. Oxford University Press, Oxford.

Bartlett, P.A. and Marlowe, C.K. (1987) Evaluation of intrinsic binding energy from a hydrogen bonding group in an enzyme inhibitor. *Science* **235**, 569–571.

Bohm, H.-J. (1994) The development of a simple empirical scoring function to estimate the binding constant for a protein ligand of known 3-dimensional structure. *J. Computer-Aided Mol. Des.* **8**, 243–256.

Bohm, H.-J. (1998) Prediction of binding constants of protein ligands: a fast method for the prioritization of hits obtained from de novo design or 3D database search. *J. Computer-Aided Mol. Des.* **12**, 309–323.

Boström, J., Norrby, P.-O., and Liljefors, T. (1998) Conformational energy penalties of protein-bound ligands. *J. Computer-Aided Mol. Des*. **12**, 383–396.

Dauber-Osguthorpe, P., Maunder, C.M., and Osguthorpe, D.J. (1996) Molecular dynamics: deciphering the data. *J. Computer-Aided Mol. Des*. **10**, 177–185.

El Tayar, N, Carrupt, P.-A., Van de Waterbeemd, H., and Testa, B. (1988) Modelling of b-adrenoceptors based on molecular electrostatic potential studies of agonists and antagonists. *J. Med. Chem*. **31**, 2072–2081.

Fersht, A.R., Shi, J.-P., Knill-Jones, J., Lowe, D.M., Wilkinson, A.J., Blow, D.M., Brick, P., Carter, P., Waye, M.M.Y., and Winter, G. (1985) Hydrogen bonding and biological specificity analysed by protein engineering. *Nature* **314**, 235–238.

Fick, A. (1855) On diffusion. Ann. Phys. **94**, 59–86.

Fleming, I. (1976) *Frontier Orbitals and Organic Chemical Reactions*. John Wiley & Sons, New York.

Goodfellow, J.M., Ed. (1991) *Molecular Dynamics: Applications in Biology*. CRC Press, Boca Raton, FL.

Goodfellow, J.M. and Moss, D.S. (1992) *Computer Modelling of Biomolecular Processes*. Ellis Horwood, Chichester, U.K.

Griffin, B.W. and Peterson, J.A. (1972) Camphor binding by *Pseudomonas putida* cytochrome P-450. Kinetics and thermodynamics of the reaction. *Biochemistry* **11**, 4740–4746.

Hadzi, D., Hodoscek, M., Kocjan, D., Solmajer, T., and Avbelj, F. (1984) The molecular electrostatic potential as a determinant of receptor-drug recognition. *Croat. Chem. Acta* **57**, 1065–1074.

Hammett, L.P (1940) *Physical Organic Chemistry*. McGraw-Hill, New York.

Hansch, C., Leo, A., and Taft, R.W. (1991) A survey of Hammett substituent constants and resonance and field parameters. Chem. Rev. **19**, 165–195.

Herzberg, G. (1970) The dissociation energy of the hydrogen molecule. *J. Mol. Spectrosc*. **33**, 147–168.

Jackson, T., Sanders, P.G., Martin, A.C.R., Lewis, D.F.V., and Morris, B.A. (1992) The molecular structure of a testosterone antibody Protein Eng. **5**, 191–261.

Jaffé, H.H. (1953) A re-examination of the Hammett equation Chem. Rev. **53**, 191–261.

Kier, L.B. (1971) *Molecular Orbital Theory in Drug Research*. Academic Press, New York.

Kolos, W. and Wolniewicz, L. (1968) Improved ground-state energy of the hydrogen molecule. *J. Chem. Phys*. **49**, 404–410.

Lengauer, T. (1997) The FlexX approach: an alternative for receptor-ligand docking and computing crystal conformations. In *Computer-Assisted Lead Finding and Optimization*. van de Waterbeemd, H., Testa, B., and Folkers, G., Eds. Wiley-VCH, New York. pp. 397–420.

Lewis, D.F.V. (1981) Ph.D. Thesis, University of Surrey.

Lewis, D.F.V. (1992) Computer-assisted methods in the evaluation of chemical toxicity. Rev. Computat. Chem. **3**, 172–222.

Lewis, D.F.V. and Griffiths, V.S. (1987) Molecular electrostatic potential energies and methylation of DNA bases. *Xenobiotica* **17**, 769–776.

Lybrand, T.P. (1990) Computer simulation of biomolecular systems using molecular dynamics and free energy perturbation methods. *Rev. Computat. Chem*. **1**, 295–320.

McCammon, J.A. and Harvey, S.C. (1987) *Dynamics of Proteins and Nucleic Acids*. Cambridge University Press, Cambridge.

Maitland, G.C., Rigby, M., Smith, E.B., and Wakeham, W.A. (1981) *Intermolecular Forces: Their Origin and Determination.* Clarendon Press, Oxford.

Nakayama, A. and Richards, W.G. (1987) A quantum chemical study of insect juvenile hormone mimics: the active confirmation and the electrostatic similarities. *Quant. Structure-Activity Relat.* **6**, 153–157.

Pettitt, B.M. and Karplus, M. (1986) Interaction energies: their role in drug design. In *Molecular Graphics and Drug Design*. Burgen, A.D.V., Roberts, G.C.K., and Tute, M.S., Eds. Elsevier, Amsterdam. pp. 75–113.

Politzer, P. and Truhler, D.G., Eds. (1981) *Chemical Applications of Atomic and Molecular Electrostatic Potentials*. Plenum, New York.

Politzer, P. and Lawrence, P.R. (1984) Relationships between the electrostatic potential, epoxide hydrase inhibition and carcinogenicity for some hydrocarbon and halogenated hydrocarbon epoxides. *Carcinogenesis* **5**, 845–848.

Politzer, P., Lawrence, P.R., and Jayasuriya, K. (1985) Molecular electrostatic potentials: an effective tool for the elucidation of biochemical phenomena. *Environ. Health Perspect*. **61**, 191–202.

Politzer, P. and Murray, J.S. (1991) Molecular electrostatic potentials and chemical reactivity. Rev. Computat. Chem. **2**, 273–312.

Pople, J.A. and Beveridge, D.L. (1970) *Approximate Molecular Orbital Theory*. McGraw-Hill, New York.

Renugopalakrishnan, V., Carey, P.R., Smith, I.C.P., Huang, S.-G., and Storer, A.C. (1991) *Proteins: Structure, Dynamics and Design*. ESCOM, Leiden.

Richards, W.G. (1983) *Quantum Pharmacology*. 2nd ed. Butterworths, London.

Rigby, M., Smith, E.B., Wakeham, W.A., and Maitland, G.C. (1986) *The Forces Between Molecules*. Clarendon Press, Oxford.

Scrocco, E. and Tomasi, J. (1973) The electrostatic molecular potential as a tool for the interpretation of molecular properties. *Top. Curr. Chem*. **42**, 95–170.

Simon, Z., Motoc, I., and Chiriac, A. (1993) Molecular basis of receptor-ligand interactions. In *Specific Interactions and Biological Recognition Processes*. Voiculetz, N., Motoc, I., and Simon, Z., Eds. CRC Press, Boca Raton, FL. pp. 17–79.

Sjoberg, P., Murray, J.S., Brinck, T., Evans, P., and Politzer, P. (1990) The use of electrostatic potential at the molecular surface in recognition interactions: dibenzo-p-dioxins and related systems. *J. Mol. Graphics* **8**, 81–85.

Sjoberg, P. and Politzer, P. (1990) The use of the electrostatic potential at the molecular surface to interpret and predict nucleophilic processes. *J. Phys. Chem*. **94**, 3959–3961.

Taft, R.W. (1956) *Steric Effects in Organic Chemistry*. John Wiley & Sons, New York.

Taft, R.W. (1960) Sigma values from reactivities. *J. Phys. Chem*. **64**, 1805–1815.

Tintelnot, M. and Andrews, P.R. (1989) Geometries of functional group interactions in enzyme-ligand complexes: guide for receptor modelling. *J. Computer-Aided Mol. Des*. **3**, 67–84.

van Gunsteren, W.G. and Weiner, P.K., Eds. (1989) *Computer Simulation of Biomolecular Systems*. ESCOM, Leiden.

Venanzi, T.J. and Venanzi, C.A. (1988) Ab initio molecular electrostatic potentials of perillartine analogues: implications for sweet-taste receptor recognition. *J. Med. Chem*. **31**, 1879–1885.

## APPENDIX

### A Derivation of Solvation and Desolvation Energies

It is possible to express the solvation energy[39] in terms of electronic, polarization, and cavity components, i.e.:

$$E_{solv.} = E_{elec.} + E_{pol.} + E_{cav.} \tag{70}$$

where each term can be evaluated as follows:

$$E_{solv.} = -\frac{1}{2}\alpha_S\mu_D^2 - \frac{k\alpha_D\alpha_S I_D I_S}{(I_D + I_S)} + k'd^2\gamma \tag{71}$$

where $\alpha$ = polarizability, $\mu$ = dipole moment, k and k′ = constants, I = ionization energy, D refers to solute, S refers to solvent, $\gamma$ = surface tension, and d = diameter.

The difference in solvation energies between an organic solvent and water is then equated with the solute partitioning energy, $\Delta E_{part}$. Therefore:

$$E_{solv.(org.)} - E_{solv.(aq.)} =$$
$$-\frac{1}{2}\alpha_O\mu_D^2 + \frac{1}{2}\alpha_A\mu_D^2 - \frac{k\alpha_O\alpha_D I_O I_D}{(I_D + I_S)} + \frac{k\alpha_O\alpha_D I_A I_D}{(I_A + I_D)} + k'd^2\gamma_o - k'd^2\gamma_A \tag{72}$$

where O refers to the organic layer and A refers to the aqueous layer.

This expression then rearranges to give the partitioning energy change, $\Delta E_{part}$:

$$\therefore \Delta E_{part.} = \frac{\mu_D^2}{2}(\alpha_A - \alpha_O) + k\alpha_D I_D\left[\frac{\alpha_A I_A}{(I_D + I_A)} - \frac{\alpha_O I_O}{(I_D + I_O)}\right] + k'd^2(\gamma_O - \gamma_A) \tag{73}$$

For n-octanol and water (if one wishes to derive, for example, a log $P_{oct}$ expression), the relevant data are as follows:

- $\alpha_{H_2O} = 1.48 \times 10^{-24}$ cm$^3$
- $\gamma_{H_2O} = 7.2 \times 10^{-2}$ N.m$^{-1}$
- $E(HOMO)_{H_2O} = 12.6$ eV
- $\alpha_{octanol} = 16.4 \times 10^{-24}$ cm$^3$
- $\gamma_{octanol} = 2.7 \times 10^{-2}$ N.m$^{-1}$
- $E(HOMO)_{octanol} \cong 10.0$ eV

and it is also possible to derive an approximation for the cavity diameter, assuming that the solute molecule is a sphere, by the following:

$$\text{area of cavity} = 4\pi\left[\frac{d}{2}\right]^2$$

$$\text{volume of cavity} = \frac{4\pi}{3}\left[\frac{d}{2}\right]^3$$

$$\therefore\ \text{area} = 3(\text{molar volume})^{2/3}$$

$$\text{and diameter}^2 = 4\left(\frac{0.75\ \text{molar volume}}{\pi}\right)^{2/3}$$

Substituting these values into the above expression provides an approximate form of the partitioning energy change, $\Delta E_{part}$, as follows:

$$\therefore \Delta E_{part} = \frac{-15}{2}\times 10^{-24}\mu_D^2 + k.I_D\alpha_D - 4.5\times 10^{-2}k'3(MV)^{2/3} \quad (74)$$

Now, the partition energy between n-octanol and water is related to the logarithm of the partition coefficient, P, as follows:

$$\log P_{oct} = \frac{\Delta E_{part}}{2.303\ RT} + \text{constant} \quad (75)$$

Consequently, by combining the previous two equations, it can be appreciated that log $P_{oct}$ is the summation of three terms relating to the molecule's dipole moment, polarizability, ionization energy/E(HOMO), and molecular size or molar volume.

It has been shown[21] that log $P_{oct.}$ values for structurally diverse small molecules correlate closely (r = 0.91) with a combination of $\alpha$, $\mu$, and E(HOMO), using an expression of the following type:-

$$\begin{array}{llll} \log P_{oct.} = 0.40\alpha_{MOL} & -\ 0.46\mu & +\ 0.33E(HOMO) - 6.06 & \\ \quad\quad\quad\quad (\pm 0.06) & \quad (\pm 0.10) & \quad\quad (\pm 0.11) & \\ n = 37; & s = 0.696; & r = 0.909; & F = 52.1 \end{array} \quad (76)$$

This expression exhibits some degree of similarity with the equation for $\Delta E_{part}$, because the $\alpha_{MOL}$ parameter correlates with MV, E(HOMO) is equivalent to $I_D$, and the dipolar terms are analogous (as $\mu$ and $\mu^2$ are obviously interrelated), although it should also be recalled that permanent molecular polarizability[40] is directly proportional to the square of dipole moment (i.e., $\alpha_{perm.} = \mu^2/3kT$, where k is the Boltzmann constant).

It is possible that, depending on the molecule, these three terms make a greater or lesser contribution to the overall log P value, and the effect of dissociation for acidic or basic compounds should also be taken into account. This can be appreciated when one considers the case of observed log P values at a given pH and also in comparisons with the log $D_{7.4}$ values of ionizable compounds[41].

Furthermore, depending on the nature of the series of chemicals under investigation, one or more of the above terms may also correlate with activity. For example, Mohammad and coworkers[42] reported a correlation with the mutagenicity of polyaromatic hydrocarbons (PAHs) which involved a combination of $\Delta E$ and $V^{2/3}$, where $\Delta E$ is the difference between the frontier orbital energies E(LUMO) and E(HOMO), and $V^{2/3}$ is the cube root of the square of molar volume. As the $\Delta E$ parameter includes E(HOMO), which is equivalent to the ionization energy of the molecule, it would appear that this QSAR with mutagenicity is essentially similar to the $\Delta E_{part}$ expression, where the $m^2$ term may be assumed to be relatively insignificant. In fact, the dipole moments of most PAHs are close to zero, and therefore it is expected that their $m^2$ terms in $\Delta E_{part}$ should be generally negligible. However, for a small number of PAHs, it is possible to show[43] that their carcinogenicity can be described in terms of a combination of $\mu^2$ and log P values. The possible reason for the appearance of a $\mu^2$ term in this instance could be due to the fact that the relatively low carcinogenic potency of some PAHs can be explained by their unusually high $\mu^2$ values. One way of rationalizing

**TABLE 3**
**Hydrogen Bonds in Various Systems†**

| | Donor | Acceptor | ΔH (kJmol$^{-1}$) | ΔH (kcal.mole$^{-1}$) | Average Value (kcal.mole$^{-1}$) |
|---|---|---|---|---|---|
| Oxygen-Oxygen | | | | | |
| O-H...O=C⟨ | Phenol | Acetone | -19.5 | -4.66 | -4.64 kcal.mol$^{-1}$ |
| | Cholesterol | Glyceryltriacetate | -18.0 | -4.30 | |
| O-H...O⟨ | Water | Dioxan | -14.5 | -3.47 | |
| | Phenol | Dioxan | -21.0 | -5.02 | |
| | Phenol | Dibutylether | -24.0 | -5.74 | |
| Oxygen-Nitrogen | | | | | |
| O-H...N⟨ | Phenol | Triethylamine | -35.0 | -8.37 | -7.41 kcal.mol$^{-1}$ |
| | Phenol | Pyridine | -27.0 | -6.45 | |
| O-H...N⟨⟨ | γ-Butyrolactam | γ-Butyrolactam | -14.5 | -3.47 | -3.94 kcal.mol$^{-1}$ |
| | α-Pyridone | α-Pyridone | -18.5 | -4.42 | |
| N-H...O=C⟨ | Aniline | Tetrahydrofuran | -12.5* | -2.99 | |
| N-H...O⟨ | Aniline | Aniline | -7.0* | -1.67 | |
| | Indole | Pyridine | -15.0 | -3.59 | -3.41 kcal.mol$^{-1}$ |
| | Pyrrole | Pyridine | -13.5 | -3.23 | |
| Nitrogen-Nitrogen | | | | | |
| O-H...N⟨ | | | | | |
| N-H...N⟨⟨ | | | | | |
| Nitrogen-Sulphur | | | | | |
| S-H...N⟨⟨ | Thiophenol | Pyridine | -10.0 | -2.39 | -3.08 kcal.mol$^{-1}$ |
| N-H...S=C⟨⟨ | γ-Thiobutyrolactam | γ-Thiobutyrolactam | -12.0 | -2.87 | |
| | α-Thiopyridone | α-Thiopyridone | -14.5 | -3.47 | |
| N-H...S⟨ | Thiocyanic acid | n-Butyl sulphide | -15.0 | -3.59 | |
| π Oxygen, Nitrogen‡ | | | | | |
| O-H...π | Phenol | Hexamethyl benzene | -7.0 | -1.67 | -1.67 kcal.mol$^{-1}$ |
| N-H...π | Aniline | Benzene | -7.0* | -1.67 | |

the appearance of a $\mu^2$ term in QSARs could involve consideration of the polarization effect as mentioned above, where the polarizability, α, of the molecule possessing a permanent dipole moment, m, is given by the expression:

$$\alpha = \mu^2 / 3kT \tag{77}$$

where k is the Boltzmann constant and T is the absolute temperature.

Additionally, the product of ionization energy and polarizability, which makes up the second term in the $\Delta E_{part}$ expression, can be regarded as representing a measure of the dispersion (or London) forces which themselves form a component of the overall van der Waals forces[37] of attraction between two molecular species. This product (I.a) also features in some QSAR expressions, such as those for melatonin analogues[44], thus indicating that dispersion forces could be involved in melatonin receptor binding. Furthermore, it is possible to make a reasonable estimate of the binding affinity between melatonin and its receptor based on the Williams et al.[45] expression for the various components likely to be involved, with hydrogen bonding (see Table 3 for some typical values) making a significant contribution[46]. In contrast, a derivation for the binding energy of camphor to cytochrome $P450_{cam}$ indicates that hydrophobic forces (desolvation) make the largest contribution[34]. Moreover, one can also employ a combination of the above quantities (such as MV and ΔE) to discriminate between different types of P450 substrates[47]. Consequently, the generalized treatment of binding energies can readily give rise to structural parameters which may be used to rationalize the differences in biological activity shown by series of related chemicals, thus leading to an understanding of the forces involved in drug-receptor binding affinities[48].

## BIOSKETCH

**David Lewis** (B.Sc., M.Sc., Ph.D.) is currently Reader in Molecular Toxicology at the School of Biomedical and Life Sciences, University of Surrey, U.K. His research interests include the Cytochromes P450, nuclear receptors, molecular modeling, QSARs and drug metabolism. Dr. Lewis is the author of two books on cytochromes P450 and he has published over 150 scientific papers.
